# Ocular Microbiota and Intraocular Inflammation

**DOI:** 10.3389/fimmu.2020.609765

**Published:** 2020-12-23

**Authors:** Jing Jing Li, Sanjun Yi, Lai Wei

**Affiliations:** State Key Laboratory of Ophthalmology, Zhongshan Ophthalmic Center, Sun Yat-sen University, Guangzhou, China

**Keywords:** ocular microbiota, ocular surface microbiome, intraocular inflammation, eye, ocular inflammatory disease

## Abstract

The term ocular microbiota refers to all types of commensal and pathogenic microorganisms present on or in the eye. The ocular surface is continuously exposed to the environment and harbors various commensals. Commensal microbes have been demonstrated to regulate host metabolism, development of immune system, and host defense against pathogen invasion. An unbalanced microbiota could lead to pathogenic microbial overgrowth and cause local or systemic inflammation. The specific antigens that irritate the deleterious immune responses in various inflammatory eye diseases remain obscure, while recent evidence implies a microbial etiology of these illnesses. The purpose of this review is to provide an overview of the literature on ocular microbiota and the role of commensal microbes in several eye diseases. In addition, this review will also discuss the interaction between microbial pathogens and host factors involved in intraocular inflammation, and evaluate therapeutic potential of targeting ocular microbiota to treat intraocular inflammation.

## Introduction

As the term microbiota refers to all types of microorganisms present in or on human body, the term ocular microbiota refers to all types of microorganisms present in or on the eyes. Ever since the launch of the Human Microbiome Project, our understanding toward the diversity and composition of commensal microbiota has been expanding. It has been evidenced that commensal microbiota plays fundamental roles in regulating host physiology, induction and development of immune system, as well as host defense against pathogen invasion, albeit dysbiosis (unbalanced microbiota) could lead to pathogenic microbial overgrowth and cause local or systemic inflammation (1). The ocular surface is directly exposed to the external environment and endangered by various pathogenic microorganisms. These facts combined with our recent findings of intraocular microbes raise intense research interest in clarifying the role of ocular microbiota in ocular health and diseases.

The ocular immune system is composed of a complex network of innate and adaptive components. Infection or autoimmunity could lead to intraocular inflammation which is associated with a multitude of sight-threatening diseases, which include but may not be limited to endophthalmitis, uveitis, age-related macular degeneration (AMD), glaucoma, and diabetic retinopathy (DR). Intraocular inflammation exerts deleterious effects on vision integrity since the delicate ocular components, such as the retina and the cornea, are unable to regenerate. The eye is an immunologically privileged organ. Up to date, the specific antigens that irritate the deleterious immune responses in the immune privileged site in those diseases remain obscure. A growing body of research has shown that commensal microbes might be the trigger of intraocular inflammation. Here, we discuss evidence for the relationship between microbes and ocular diseases, discuss microbial pathogen and host factors, including the molecular and cellular interactions, involved in non-infectious intraocular inflammation, and evaluate therapeutic potential of targeting ocular microbiota to treat intraocular inflammation.

## The Ocular Microbiota

The ocular surface is the interface between the eye and the environment which comprises the cornea, the conjunctiva, the tear film, and the eyelids. It has been much debated whether the microorganisms in our environment are able to adhere to and colonize the ocular surface, because during eye blinking, tears secreted from the lacrimal gland of the eyes contain lysozyme that could kill bacteria and wash the ocular surface (1). Supporting evidence for the existence of ocular surface microorganisms arises from microbial cultivation studies documented first in 1930 (2). Many subsequent results from similar studies are in line with the first discovery. Swabs from different parts of the ocular surface were incubated in bacteria growth media (mostly blood and chocolate agar). The incubations occur in aerobic, anaerobic, or 5% CO_2_ for up to 14 days at body temperature (3). These culture-based methods are invaluable in a historical perspective in confirming the existence of a microbiota and identifying microorganisms. The common bacteria isolated from these sites of the eye are Gram-positive genera, including coagulase-negative *Staphylococcus*, *Streptococcus*, *Propionibacterium*, *Diphtheroid bacteria* and *Micrococcus* (4). Some genera that are abundant in the gut flora, such as *Escherichia, Enterococcus*, *Lactobacillus*, and *Bacillus* are less common on the normal ocular surface (4). Gram-negative genera, such as *Haemophilus*, *Neisseria*, *Pseudomonas*, and fungal isolates are even rarer but can also be isolated and cultured from the surface of eyes without obvious signs of inflammation or infection (4, 5). The most common ocular surface bacteria are coagulase-negative *Staphylococci* which present in 20–80% of the swabs from the conjunctiva and 30–100% of the swabs from the lids (4). The density of microbes recovered are usually lowest from tears while higher from conjunctiva and eye lid (3). Types of the ocular surface microbes identified are consistent with results from studies of cultivable microbiota from contact lenses, which also suggest that coagulase-negative *Staphylococcus* is the most common genera, and less commonly *Bacillus, Micrococcus*, and fungi (3).

**Figure 1:**
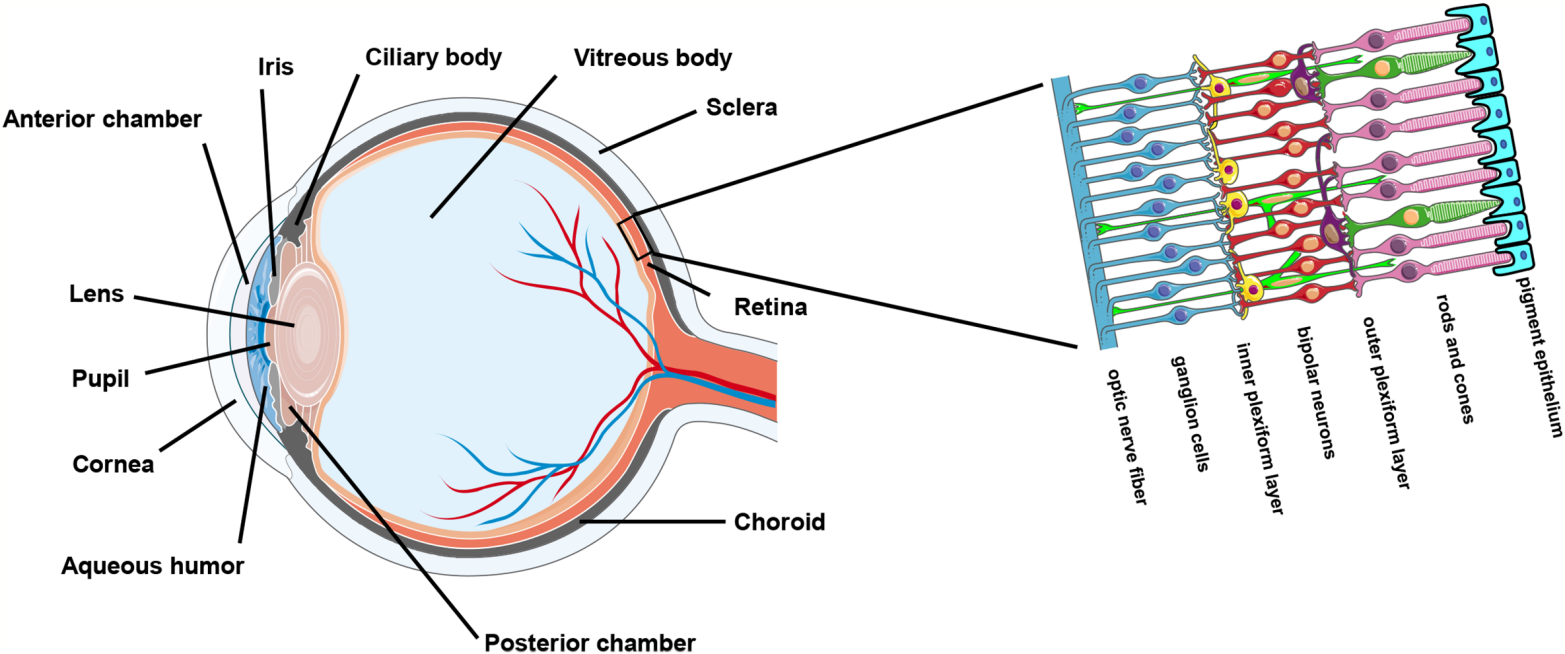
Anatomy of the eye.

The ocular surface and the sclera enwrap the interior ocular cavity. The cavity enclosed by the ocular outer compartments mainly consists of the anterior chamber, the posterior chamber, the ciliary body, and the vitreous body (**Figure 1**). Historically, the intraocular environment has been deemed sterile on account of its closed anatomical structure as well as protection provided by the tight and restrictive blood-retina barrier unless it is invaded by pathogens due to unnatural circumstances. Contamination could occur when the outer ocular compartments are damaged during an intraocular surgical procedure or following an injury caused by a penetrating foreign object (6, 7). For example, post-operative infectious endophthalmitis is a rare but serious vision-threatening complication of ocular surgery (e.g., cataract extraction) which involves inflammation of both the anterior and posterior segments of the eye. Post-operative endophthalmitis is presumably attributed to the diffusion of microorganisms from the patient’s conjunctival or skin flora into the sterile intraocular compartments of the eye during surgery and cause overwhelming inflammation (8). Besides, diseased conditions that are associated with retinal vascular lesions could introduce microbial invasions from the circulating blood as normal human blood contains appreciable numbers of microorganisms (9–12). For example, one of the early pathological features of DR, a common diabetes complication in the eye, is retinal vascular leakage (13). Coincidently, DR has been demonstrated to be affected by microbiome (14, 15). Furthermore, some microbes (mainly viruses) could spread along the nerves. Cases of infections occurred in the central nervous system (CNS) by neurological spread are not sparse. Ocular infection could be secondary to a CNS infection. For instance, it has been reported that rabies virus infected in the hind limbs of mice could travel along the peripheral nerve and the axon to the brain and then spread to the eye through the optic nerve where it infects the retinal ganglion cell (RGC) but not the photoreceptors (16).

The vitreous humor and aqueous humor contain a variety of organic and anorganic components that form an excellent cultivating medium for microorganisms (17, 18). In the late 19th century, researchers found that microorganisms, such as *Bacillus Subtilis* and *Bacillus Megaterium* grew extremely well in the aqueous humor withdrawn from living body (18). In some studies by others, *Propionibacterium acnes* (*P. acnes*) was detected in the granuloma of the retina in patients with ocular sarcoidosis where accumulated CD4^+^ cells and CD68^+^ cells were also nearby, suggesting that *P. acnes* could be associated with sarcoid uveitis (19, 20). In line with their findings, we were able to detect the expression of *P. acnes* mRNA in most aqueous humor specimens we collected from patients undergoing cataract surgeries who were free of active or history of intraocular inflammation and infection, raising the question of whether *P. acnes* is a benign resident or a pathogenic intraocular microorganism and whether there is a community of microorganisms living inside the human eye. So far, there is no direct documentation of the existence of intraocular microbiome. This is possibly because the intraocular materials from healthy human eye are difficult to acquire. In our preliminary study, the intraocular microbial communities were significantly different among patients with distinct ocular diseases. Whether the intraocular microbiome lives in symbiosis with the host just as the intestinal microbiome and whether alteration of intraocular microbiome contributes to the ocular health and the etiology of ocular diseases in general remain to be examined.

## Defining The Ocular Microbiota

Methods to define a microbiota can be generally divided into culture-based techniques and non-culture-based techniques. The culture-based techniques depend on phenotypic characteristics of microbes to estimate the microbial load, for example, the ability of microbes in a sample to proliferate in or on a specified growth medium under a specified growth condition (21, 22). Although it provides a rough evaluation of microbial density and diversity in specimens, these measures are often inaccurate and biased. The cultivable species may only represent a small proportion of the real microbial populations in the samples which are prone to grow under the applied cultivation conditions (23, 24). In addition, the estimation of microbial density in a certain sample also varies according to a wide range of factors that may affect the proliferation ability of microbes. Some microbes are even uncultivable on traditional laboratory medium. Currently, only half of the bacterial phyla have cultivated representatives (25). Indeed, variations in types and density of microorganisms that can be cultured from the ocular surface exist in many published studies (23, 26).

The more advanced non-culture diagnostic methods are immunoassays, which target microbe-secreted peptides or microbial antigen, and metagenomic sequencing, which target microbial RNA or DNA. Both methods allow study of the community of the microbes present without obtaining pure cultures. Methods targeting microbial nucleic acids do not require specific antibodies making them more readily available for laboratory study. 16S ribosomal RNA (rRNA) is commonly used for taxonomic purposes for bacteria, while 18S rRNA and internal transcribed spacer (ITS) are used for fungi. To define microbial species, the 16S/18S/ITS gene amplicons are usually sequenced and the sequence will be matched with the repository of existing sequence to yield taxonomic information. Nowadays, more than 9,000 16S rRNA gene sequences have been deposited in GenBank, rendering 16S rRNA gene sequence analysis a better tool to identify those rarely isolated, poorly described, and uncultivable bacteria (27). Another sequencing method called shotgun metagenomics can achieve species- and strain-level resolution. It examines the entire genome as opposed to only the 16S/18S/ITS amplicons, but its high costs and heavy demands on bioinformatic analyses precluded its extensive use for microbiome study (28).

The first high-throughput study to explore the diversity of healthy human ocular surface microbiome was published in 2007 by Graham et al. in which they identified *Staphylococcus*, *Rhodococcus*, *Corynebacterium*, *Propionibacterium*, *Klebsiella*, *Bacillus*, and *Erwinia* as the main bacterial genera on healthy human ocular surface (2). However, the composition of the “core ocular surface microbiota” has been highly contested. Dong et al. proposed that 12 genera—*Pseudomonas*, *Propionibacterium*, *Bradyrhizobium*, *Corynebacterium*, *Acinetobacter*, *Brevundimonas*, *Staphylococcus*, *Aquabacterium*, *Sphingomonas*, *Streptococcus*, *Streptophyta*, and *Methylobacterium*—represented the putative “core” of conjunctival microbiota (29). Another study claimed that *Corynebacterium*, *Streptococcus*, *Propionibacterium*, *Bacillus*, *Staphylococcus*, and *Ralsontia* were detected in 80% of the 105 samples tested and together accounted for more than a third of the entire bacterial community characterized (30). The core conjunctival microbial communities were later shown to be composed of *Corynebacterium*, *Pseudomonas*, *Staphylococcus*, *Acinetobacter*, *Streptococcus*, *Millisia*, *Anaerococcus*, *Finegoldia*, *Simonsiella*, and *Veillonella* (31). Doan et al. applied three different techniques to explore the healthy human conjunctiva microbiome: bacterial culture, 16S rDNA gene deep sequencing, and biome representational in silico karyotyping. They found that *Corynebacteria*, *Propionibacterium*, and coagulase-negative *Staphylococcus* were the predominant organisms (32). A study by Ozkan et al. reported that *Corynebacterium*, *Acinetobacteria*, *Pseudomonas*, *Sphingomonas*, *Streptococcus*, *Massilia*, and *Rothia* accounted for 80% of the operational taxanomic units (OTUs) and microbial genera on the ocular surface (24). The metagenomic data collected from our laboratory revealed that *Propionibacterium*, *Staphylococcus*, *Escherichia*, and *Micrococcus* were the most abundant ocular surface microbial genera in healthy humans (33). Li et al. found the predominant genera to be *Pseudomonas*, *Acinetobacter*, *Bacillus*, *Chryseobacterium*, and *Corynebacterium* (34). The more recent study carried out by Suzuki et al. demonstrated that the ocular surface was typically dominated by *Propionibacterium* in the young subjects and by *Corynebacterium* or *Neisseriaceae* in the elderly subjects (35).

Although metagenomic sequencing offers substantial information about the diversity of the ocular microbiome and reveal previously unidentified microbial species by the traditional culture-based methods, inconsistency remains between several studies using similar sequencing techniques (**Table 1**). Therefore, culture-based methods are sometimes combined with non-culture-based methods to prove the existence of a microbe. Most of the metagenomic sequencing results support *Corynebacterium*, *Propionibacterium*, and *Staphylococcus* as the dominant taxons of healthy ocular surface. This expands the list of the most common genera recovered by culture-based methods, *i.e.* the coagulase-negative *Staphylococcus*. Noteworthy, metagenomic sequencing results could also be complicated by several factors, such as small sample size (36), depth of swabs (37, 38), and contaminations from DNA extraction kit and PCR reagents (39, 40) and so forth. Nonetheless, it remains the best state-of-the-art tool for *in situ* profiling of a microbiota. Utilizing this powerful metagenomic sequencing tool, we have also characterized the intraocular microbiota of the aqueous humor from patients with ocular diseases that required surgical intervention. We found that each disease has a unique intraocular microbial signature, suggesting a potential link between intraocular microbiota and ocular health and diseases.

**Table 1:** Core ocular surface microbiome in healthy adults.

Genera of bacteria	Number of samples	Reference
*Staphylococcus*, *Bacillus*, *Rhodococcus*, *Corynebacterium*, *Propionibacterium*, *Klebsiella*, and *Erwinia*	57	(2)
*Pseudomonas*, *Propionibacterium*, *Bradyrhizobium*, *Corynebacterium*, *Acinetobacter*, *Brevundimonas*, *Staphylococcus*, *Aquabacterium*, *Sphingomonas*, *Streptococcus*, *Streptophyta*, and *Methylobacterium*	4	(29)
*Corynebacterium*, *Streptococcus*, *Propionibacterium*, *Bacillus*, *Staphylococcus*, and *Ralsontia*	105	(30)
*Corynebacterium*, *Pseudomonas*, *Staphylococcus*, *Acinetobacter*, *Streptococcus*, *Millisia*, *Anaerococcus*, *Finegoldia*, *Simonsiella*, and *Veillonella*	31	(31)
*Corynebacteria*, *Propionibacteria*, and *Staphylococcus* *Corynebacterium*, *Acinetobacteria*, *Pseudomonas*, *Sphingomonas*, *Streptococcus*, *Massilia*, and *Rothia* *Propionibacterium*, *Staphylococcus*, *Escherichia*, and *Micrococcus* *Pseudomonas*, *Acinetobacter*, *Bacillus*, *Chryseobacterium*, *and Corynebacterium* *Propionibacterium*, *Corynebacterium*, and *Neisseriaceae*	107 45 90 54 36	(32) (24) (33) (34) (35)

## Factors Changing The Ocular Microbiota

The ocular surface microbiota can be influenced by environmental conditions, age, gender, personal habits, contact lens wear, disease states, antibiotics, and infection etc (41). Understanding toward the factors that alter the intraocular microbiota is still in its infancy. As the intraocular space is relatively separated from the outer environment, it is reasonable to speculate that the intraocular microbiota is more imaginably associated with host factors.

Age and sex hormone have significant impacts on immune regulation and ocular health (33). Our study showed that age groups differs significantly in bacterial composition and metabolic functions, and that gender factor only affects β but not α diversity of bacterial composition. Our data suggest that age and gender can collectively shape the ocular surface microbiome, while age appears to be a stronger factor in reshaping the ocular surface microbiome (33). However, some earlier studies showed contradictory findings: Ozkan et al (24). found no effect of age on the microbial a diversity and a higher Shannon diversity index in males; Zhou et al. found no effect of sex on the microbial diversity and a higher richness and Shannon diversity index in children less than 10 years old (30). This inconsistency may be explained by the fact that the techniques used were different in these studies. We used metagenomic sequencing approach which may detect a much broader range of microbes (33).

Dry eye syndrome is a multi-pathogenic factorial disease of the ocular surface characterized by loss of homeostasis of the tear film which results in excessive evaporation of tears in most of the cases. Meibomian glands located in the eyelids are responsible for the secretion of oily components for the tear film to protect the ocular surface from overt dryness, discomfort, or damage. Meibomian gland dysfunction often leads to evaporative dry eye syndrome. Inconsistencies in the microbial species that changed by dry eye syndrome and meibomian gland dysfunction remain in several studies (34, 42, 43). This may again be explained by sample size, sequencing approach, and different diagnostic criteria. For example, it has been reported that some cases of meibomian gland dysfunction overlap with not only dry eye syndrome but also blepharitis (44). No conclusive results have been achieved by these analyses, yet the results hinted that the ocular surface “resident microbiota”, *Corynebacterium*, is likely associated with these diseases. However, it is still unclear that whether the change of the ocular surface microbiota is a cause or a consequence of the ocular surface disorders.

Patients with other diseases such as diabetes, high cholesterol and triglycerides, conjunctivitis, autoimmune diseases like Behcet’s disease (BD), rheumatoid arthritis, and Sjögren’s syndrome, which are linked with meibomian gland dysfunction and dry eye syndrome have also been reported with altered ocular surface microbiota (45–53). These suggest that endogenous host factors other than age and gender may be equally important in shaping the ocular surface microbiome, or the change of ocular surface microbiome may be secondary to meibomian gland dysfunction or dry eye syndrome. Further investigations are needed to dissociate data of patients with meibomian gland dysfunction or dry eye syndrome from those without in order to provide insights into finding the endogenous host factors that alter ocular surface microbiome.

## Intraocular Inflammation

**Figure 2:**
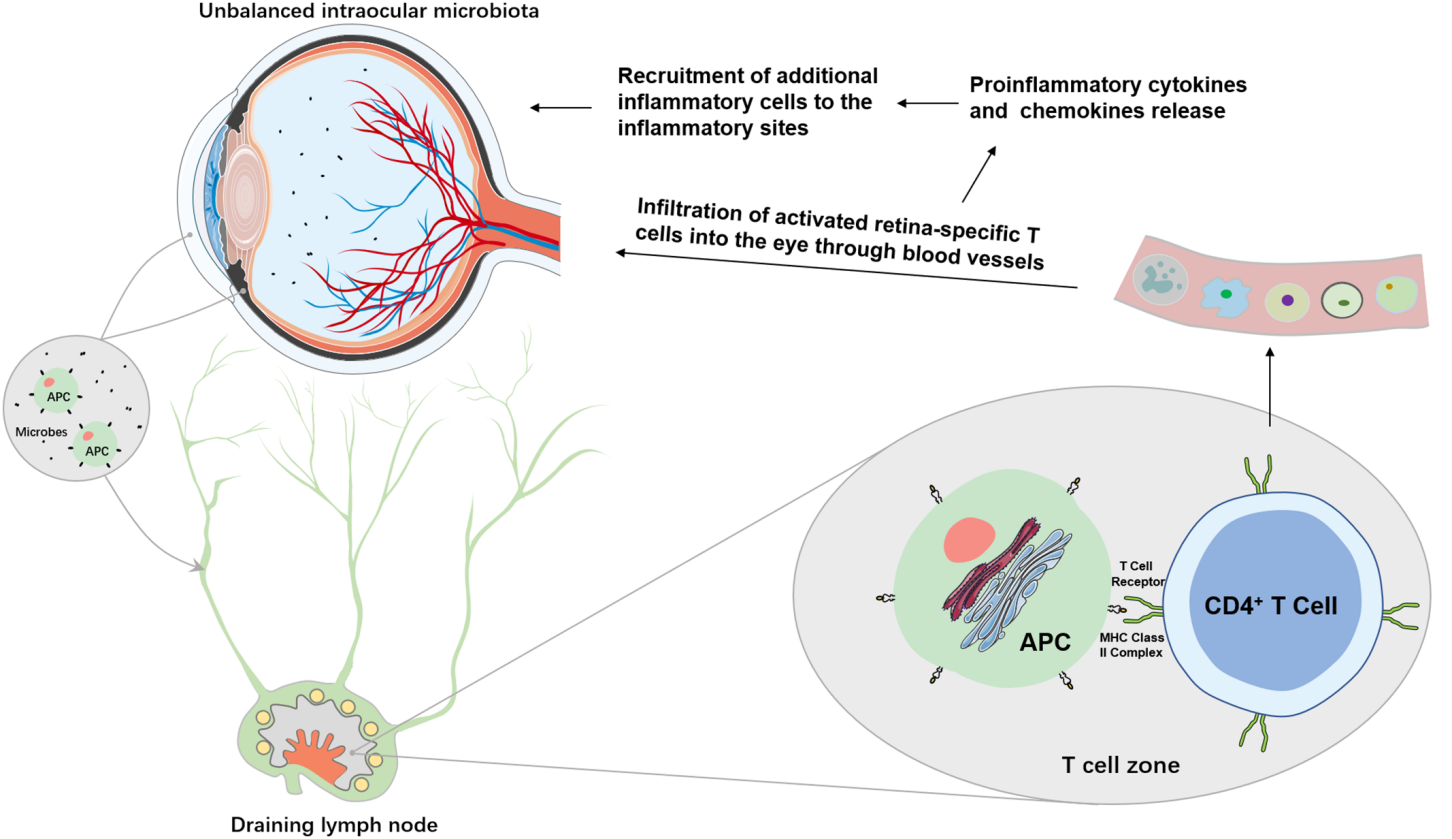
Potential mechanisms of intraocular microbiome-mediated intraocular inflammation. Unbalanced intraocular microbiota can lead to overgrowth of pathogenic microbes which are surveyed by resident ocular APCs, such as ocular DCs. Immature APCs take up either the microbe as a whole or the microbial antigens in the eye to become mature. Through afferent lymphatic vessels, mature APCs migrate to the closest draining lymph nodes where they are recognized by CD4^+^ T cells. Activated retina-specific T cells migrate into the eyes to secret proinflammatory cytokines and chemokines which may disrupt the blood-retina barrier and recruit additional inflammatory cells and mediators to the eyes to cause intraocular inflammation.

Intraocular inflammation has two types: acute and chronic. The acute intraocular inflammation as observed in post-operative or post-traumatic endophthalmitis is normally caused by pathogenic microbes and is capable of inducing robust changes in blood-retina barrier permeability and subsequent infiltration of non-resident immune cells, such as polymorphonuclear leukocytes (PMNs) and macrophages (**Figure 2**) (54). The chronic form accounting for most intraocular inflammation appears to arise from a combination of predisposing genetic and environmental factors. A break of immune tolerance against endogenous antigens, followed by autoantibody production, dysregulation of effector and regulatory T cells, and infiltration of T lymphocytes and macrophages, is usually the mechanistic basis for chronic intraocular inflammation associated with inflammatory ocular diseases, particularly uveitis.

The eye is an immunologically privileged organ. The anterior chamber, the vitreous cavity, and the subretinal space of the eyes, are immune privileged sites where multiple mechanisms work together to inhibit overt immune responses (55). The conjunctiva and the interior of the eye are highly vascularized and home to many immune cells, suggesting that the eyes are under sophisticated regulation by immune system. Presumably due to an evolutionary adaption, our immune system uses several strategies to avoid intraocular inflammation, for example, immunological ignorance of eye-derived antigens, immune tolerance, and a local immunosuppressive or anti-inflammatory microenvironment created by blood-retina barriers and immunosuppressive/anti-inflammatory molecules in ocular fluids or constitutively expressed by ocular parenchymal cells (55). These strategies are collectively known as ocular immune privilege. By virtue of ocular immune privilege, transplants into the eyes are not subjected to sight-destroying immune responses and inflammation. Antigen injected into these places induces peripheral tolerance to that antigen (56). A classic phenomenon is the anterior chamber-associated immune deviation (ACAID) in which injection of an antigen to the anterior chamber of the eye induces systemic immunoregulatory responses that are believed to protect the eye from future immune-mediated damage (57). Similarly, adeno-associated virus delivery to the subretinal space elicits only limited immune response (58, 59). However, in the face of antigen overload, the mechanisms of immune privilege cannot always effectively restrain the immune cells from infiltrating and responding. The C56BL/6 mice eyes intravitreally injected with 500 colony-forming unit (cfu) of *Staphylococcus aureus* were observed to reach 10^7^ cfu 24 hours post injection and eventually reduced the number to 5×10^3^ cfu by 72 hours. During the 3 day course, mice had few infiltrating inflammatory cells and no microscopic retinal damage. By contrast, eyes intravitreally injected with 5,000 cfu of the same microbes resulted in massive infiltration and severe damage to ocular structures (60).

## Ocular Inflammatory Diseases are Associated with Non-Ocular Microbiota

The microbiota was once considered to be inert in immune homeostasis. However, over the past decades, pivotal roles of dysbiosis (changes in the gut commensal microbiota) in human health have been established. Dysbiosis has been implicated in various diseases associated with systemic inflammation, including rheumatoid arthritis, multiple sclerosis, inflammatory bowel disease, and type 1 diabetes (61–67). In recent years, gut commensals have also been involved in the pathogenesis of several non-infectious eye diseases like autoimmune uveitis, AMD, and glaucoma.

The eye is protected by a prompt immune response to clear pathogens or antigens. However, inappropriate intraocular inflammation, such as those occurs in various forms of uveitis could be fatal to the eye and its visual function. Uveitis is a group of eye diseases characterized by acute or chronic intraocular inflammation of infectious or non-infectious origin. Autoimmune uveitis arises without known infectious stimuli. In humans, uveitis has been associated with human leukocyte antigen-B27 (HLA-B27), a prominent major histocompatibility complex (MHC) class I‐allele expressed on the white blood cell surface, suggesting innate etiology (68). Patients with acute anterior uveitis have distinct intestinal microbial signature (69). Animal models have provided tremendous insights into its mechanistic basis. HLA-B27 transgenic rats exhibited altered cecal microbiota compared to healthy controls (70). Other mouse models of induced or spontaneous uveitis also showed ameliorated inflammation when commensals were removed or reduced. A classic mouse model of autoimmune uveitis is the experimental autoimmune uveitis (EAU) in which interphotoreceptor retinoid binding protein (IRBP) and heat-killed *Mycobacterium tuberculosis* are co-administered in complete Freund’s adjuvant and ocular inflammation begins around day 10. Fecal microbiota transplantation with feces from BD patients significantly exacerbated EAU and increased the production of inflammatory cytokines (71). In EAU, an intestinal dysbiosis accompanies a disruption in intestinal homeostasis was demonstrated (72). Microbial ablation by raising mice in germ-free environment or microbial reduction by the treatment of a combination of broad-spectrum antibiotics in EAU reduce the severity of ocular inflammation (73). The direct connection between intestinal microbiota and the eye was confirmed in a spontaneous uveitis mouse model in R161H mice which are engineered to express a transgene for T cell receptor (TCR) specific for IRBP. In these mice, retina-specific T cells are first activated by intestinal commensals in the gut through the autoreactive TCR and then trigger inflammation in the retina, implying that a commensal microbial antigen may mimic a retinal antigen to trigger autoimmune uveitis (**Figure 2**) (74). In line with this evidence, another study demonstrated that broad-spectrum oral antibiotics could attenuate EAU by increasing Tregs and decreasing effector T cells in the gut and extraintestinal tissues (75).

AMD is a progressive retinal degeneration and often associated with chronic low-grade intraocular inflammation. A substantial genome-wide association studies revealed that genetic variants in complement and various inflammatory pathway such as *complement factor* (*CF*) *H*, *CFI*, *age-related maculopathy susceptibility 2* (*ARMS2*), *tissue inhibitor of metalloproteinases-3* (*TIMP3*), and *matrix metallopeptidase 9* (*MMP9*), as well as in lipid pathway such as *apolipoprotein E* (*APOE*), *lipase C* (*LIPC*), *cholesteryl ester transfer protein* (*CETP*), and *ATP-binding cassette transporter* (*ABCA1*) were associated with the disease (76, 77). The identified variants, however, explain only 46–71% of the genomic heritability of AMD (78). This may be attributed to additional variation not identified, or to genetic interaction with environmental factors such as smoking, diet or sunlight exposure (76). Commensal bacteria as one potential environmental influence could play a role in AMD development. There are several reasons for such presumption: (i) Microbes are involved in the regulation of host immunity and lipid metabolism both of which have profound effects on chronic low-grade inflammation and are important in the pathogenesis of AMD. (ii) Drusen in AMD eyes contain anti-infectious components such as complement components, apolipoprotein E, amyloid β, vitronectin, immunoglobulins and C1Q (79). (iii) As discussed earlier, gut microorganisms could send signals that exert distal effects on a remote organ like the eye. Indeed, several studies have linked commensal microbiota to AMD pathogenesis. Patients with AMD have shown distinct intestinal, oral, nasal, and pharyngeal microbial communities, highlighting potential role of mucosal surface microbes in the pathogenesis of AMD (80–82). Mice fed a high-glycemic diet developed hallmarks of AMD, such as retinal pigment epithelium (RPE) hypopigmentation and atrophy, lipofuscin accumulation, and photoreceptor degeneration, whereas mice fed the lower-glycemia diet did no (83, 84). High-fat diet feeding in mice significantly influenced gut microbiota composition and exacerbated laser-induced model of neovascular AMD, also known as choroidal neovascularization (85). In our preliminary study, we observed that several intraocular bacteria were associated specifically with AMD and significantly enriched in soft drusen from AMD patients implying that bacterial infection may be a previously unappreciated etiology of early AMD.

Gut and oral commensals have been implicated in glaucoma which involves local inflammatory response. Astafurov et al. demonstrated that patients with glaucoma had higher oral bacterial load than patients without glaucoma, and that low-dose subcutaneous lipopolysaccharide (LPS) in two separate animal models of glaucoma resulted in enhanced glaucomatous neural degeneration (86). DBA/2J mice, which spontaneously develop high intraocular pressure, mild intraocular inflammation and glaucoma by 6–8 months of age, have been frequently used as a murine model for glaucoma (87). Interestingly, DBA/2J mice raised in germ-free environment do not display typical axonal degeneration and neuronal loss at 12 months of age (88). The study demonstrated that T cells at least partially mediated the glaucomatous pathology in DBA/2J mice raised under specific-pathogen-free conditions (88). A more recent study found that compared to healthy subjects, the gut microbiota of patients with primary open-angle glaucoma had a different gut microbiota profile (89).

The molecular mechanisms underlying the altered gut microbiota and the progression of these ocular inflammatory diseases remain obscure. Further investigations are required to elucidate whether translocation of microbes and/or microbial products (e.g., LPS, peptidoglycan, short-chain fatty acids, and microbial DNA) from the gastrointestinal tract or other mucosal surfaces to the eye through blood circulation or ocular lymphatics occurs during the progression of diseases.

## Microbial Pathogens and Host Factors in Intraocular Inflammation

During an event of intraocular inflammation, the immunosuppressive environment of the eye is compromised when pathogens or antigens are detected by local immune surveillance. An immune response is usually initiated by innate receptors, such as Toll-like receptors (TLRs), located in the retina. TLRs generally recognize pathogen-associated molecular patterns (PAMPs) and damage-associated molecular patterns (DAMPs). The former comprise microbial structures/nucleic acid sequences, while the latter are usually molecules released from host cells following tissue injury or damage. The complex interactions between PAMPs/DAMPs and host immune system are mediated through multiple host factors including antigen presenting cells (APCs), MHCs, and inflammatory mediators (**Figure 2**).

### Toll-Like Receptors

The activation of an innate immune response is followed by the recognition of PAMPs on the surface of microbes by a group of receptors called pattern recognition receptors (PRRs). Of the several different families of PRRs which includes TLRs, NOD-like receptors, and mannose receptors, TLRs are the most important members and have been extensively researched in the field of intraocular inflammation. The TLRs are type 1 integral membrane receptors with an N-terminal extracellular domain for ligand binding composed of leucine rich repeats and a C-terminal cytoplasmic Toll/IL-1 receptor (TIR) domain for intracellular signaling (90). Bacterial structural components such as LPS, peptidoglycan, lipids, and lipoproteins can be detected by TLRs on RPE cells, retinal microglia, astrocytes, and Müller cells. As of today, 10 functional TLRs have been identified in humans, while 12 TLRs have been identified in mice (91). TLR1, 2, 4, 5 and 6 are expressed on the cell surface and mainly recognize PAMPs derived from bacteria, fungi and protozoa. In contrast, TLR3, 7, 8 and 9 are expressed in the cytoplasmic compartment and primarily recognize nucleic acids derived from virus and bacteria (91). Upon ligand-receptor interactions, TLRs recruit adaptor molecules including Myeloid differentiating factor 88 (MyD88) and TIR domain containing adaptor inducing interferon β (TRIF), TIR domain-containing adaptor protein (TIRAP) and TRIF-related adaptor molecule (TRAM). Stimulation of TLR signaling further induces nuclear factor kappa-B (NF-κB) nuclear translocation, or activation of interferon regulatory factors (IRFs), or mitogen-activate protein kinases (MAPKs) pathways to promote the expression of inflammatory mediators (92).

Previous studies have linked TLR signaling to inflammatory eye diseases (93–96). Significantly higher expression of TLR 2, 3, 4, and 8 has been observed in BD patients as compared with healthy controls (93). A selective perturbation in the expression and function of TLR2 and 4 was observed on the neutrophils and monocytes of patients with acute anterior uveitis (AAU) (96), suggestive of microbial triggers and TLRs in the pathogenesis of AAU, as TLR2 or TLR4 stimulation by their ligands results in internalization of the cell surface receptors (97, 98). The activation of TLR4 by endotoxin induces a standard model of uveitis in rats, referred to as endotoxin-induced uveitis (99), although the molecular mechanisms by which endotoxin induces uveitis remain unknown. Injection of different TLR agonists into iris/ciliary body explants increased production of inflammatory cytokines TNF-α, IL-6, IP-10/CXCL10, MCP-1. Intraocular injection of TLR agonist increased leukocyte interactions with the endothelium of the iris vasculature and chemotaxis into the iris tissue (100). Contradictorily, studies utilizing genetic mouse models demonstrated that TLR2, 3, 4, and 9 are highly redundant in the adjuvant effect needed to induce EAU and that diverse microbial infections may contribute to the pathogenesis of uveitis (101). TLRs may partially explain the experimental and clinical manifestations of immune-mediated uveitis implicating microbial triggers (102, 103).

### Antigen-Presenting Cells

Professional APCs include dendritic cells (DCs), macrophages and B cells. APCs express and use antigen-specific surface receptors, such as PRRs, to recognize and bind their targets, such as PAMPs or DAMPs. Binding of surface PRRs to microbial PAMPs or whole bacteria induces phagocytosis of microbial pathogens by DCs or macrophages. A previous study demonstrated that the protein expression of functional endotoxin receptor TLR4 and its associated LPS receptor complex (CD14 and MD-2) was restricted to resident APCs in the normal human uvea, consisting mainly of HLA-DR^+^ DCs. These APCs appeared to be strategically located in perivascular and subepithelial sites to detect and respond to blood-borne or intraocular LPS of Gram-negative bacteria (104). These observations were confirmed by another study showing the expression of the functional TLR4 and CD14 in human ciliary body and TLR4 in human iris endothelial cells (105), supporting the notion that microbial triggers, in particular LPS of Gram-negative bacteria may trigger AAU by activating TLR4-expressing resident uveal APCs. RPE cells *in vitro* display many characteristics typical of APC yet are poor inducers of lymphocyte proliferation (106). RPE cells express proteins for TLR1-6 and 9, and are capable of secreting IL-8 after stimulation by PAMPs (107). APCs themselves may release cytokines to directly influence the development of uveitis (108) or may pathogenically link to uveitis through regulating the function of T cells (109, 110).

### Major Histocompatibility Complexes

After binding to respective PRR ligands, APCs internalize and degrade their targets by initiating phagocytosis or clathrin-mediated endocytosis, and then display the epitope by MHCs for recognition by immunologic structures like TCRs on appropriate T cells. MHCs are cell surface proteins essential for adaptive immune response. Two types of MHCs display antigens: class I MHCs and class II MHCs. Class I MHC receptors are produced by all nucleated cells and display endogenous antigens to activate CD8^+^ cytotoxic T cells. Class II MHC receptors normally express only on professional APCs (111). HLA-B27 is present in about 50% of all patients with AAU and is the strongest genetic factor for AAU. Both HLA-B27 transgenic Lewis rats and HLA-B27 transgenic Fischer rats developed gut inflammation (112). The rats develop diarrhea, peripheral arthritis, and spondylitis, while removal of microbiota significantly reduces the symptoms, however, these rats do not develop uveitis (68, 113). Similarly, in humans, the majority of individuals who are HLA-B27-positive do not develop AAU or other autoimmune diseases, implying the involvement of other genes or environmental factors in the development of uveitis. Some literature suggests the involvement of microbial triggers in disease development, in particular Gram-negative bacteria (114, 115).

### Inflammatory Mediators

Proinflammatory mediators released by retinal microglia, endothelial, or Müller cells are critical for recruiting PMNs and macrophages to sites of inflammation or infection. In addition to pathogen clearance, PMNs and macrophages produce cytokines and chemokines to increase blood-retinal barrier permeability, facilitate migration of cells, and recruit additional inflammatory cells and mediators to the inflammatory sites. Many of the proinflammatory mediators increased in experimental animal models of intraocular inflammation are also diagnosed with increased levels in the aqueous and vitreous humor of humans with inflammatory eye diseases (116). Several inflammatory mediators play essential roles in mounting an inflammatory response. TNFα is a pleiotropic cytokine that rapidly upregulates following tissue injury and can be produced by activated macrophages, T cells, and natural killer cells (117). In the eye, TNFα appears to mediate the pathogenesis of intraocular inflammation, neovascular and retinal degeneration by stimulating other retinal cells to produce cytokines, such as interferon γ, IL-1β, IL-2, IL-4, IL-6, IL-8, and IL-10, and other proinflammatory molecules, such as vascular cell adhesion molecule-1 (VCAM-1), intercellular cell adhesion molecule-1 (ICAM-1), and macrophage inflammatory protein (MIP)-1α, MIP-2, monocyte chemotactic protein-1 (MCP-1), and CXCL1 (117–120). During an intraocular inflammation, elevated levels of TNFα correlated with worsen visual acuity (120). TNFα^-/-^ mice were subjected to greater bacterial growth, intraocular inflammation, and ocular structural damage after an infection (121). While bacterial growth was similar between wild-type controls and IL-6^-/-^ and CXCL1^-/-^ mice, the intraocular inflammation was attenuated in mice lacking CXCL-1, but not IL-6 (122).

## Targeting Ocular Microbiota as Novel Therapies for Intraocular Inflammation

Current treatment for intraocular inflammation includes steroids, anti-cytokine biologics, and non-biologic immunosuppressive agents therapies. While corticosteroids remain the most potent and efficacious drug for treating intraocular inflammation, results from the corticosteroids use are not optimal and poor control in some cases (123). Patients diagnosed with uveitis require prolonged repeated intravitreal injections which usually leads to many side effects, such as hyperglycemia and dysregulated bone metabolic homeostasis (124). The drugs most commonly used in replacement of corticosteroids are non-biologic immunosuppressive agents including azathioprine, methotrexate, mycophenolate and cyclosporine, all of which have been reported with potentials for significant side effects (123). The more recent anti-cytokine biologics have greatly changed the therapeutic options for non-infectious uveitis. In 2016, adalimumab as the first anti-TNFα biologic, was approved by the FDA in the treatment of non-infectious intermedia uveitis, posterior uveitis, and panuveitis. Similar anti-TNFα biologics, such as infliximab and golimumab, could also exert significant anti-inflammatory effects. In TNFα^-/-^ mice, the intraocular inflammation and cytokine production was dramatically reduced in the experimental model of endophthalmitis. Unfortunately, TNFα^-/-^ mice displayed poor retinal function retention, presumably due to increased bacterial load (121), providing rationale for the development of novel and more targeted therapies.

R161H mice which spontaneously developed uveitis or EAU mouse models are devoid of disease phenotypes when raised in germ-free environment or treated with broad-spectrum antibiotics (73, 74). Mechanistic basis to these phenomena has been proven by Horai et al. to be originated from gut microbe-activated T cells (74). Our recent findings of the existence of intraocular microbes imply that intraocular commensals might also contribute to the pathogenesis of uveitis, as germ-free environment and antibiotics theoretically also remove intraocular commensals, although direct evidence for this notion is still lacking. Therapies manipulating commensal microbiome have been emerged as a novel strategy to prime the host immune system to counteract several inflammatory diseases, such as inflammatory bowel disease, graft-vs.-host disease, HIV infection, and psychological-stress-induced inflammation (125–127). This is presumably due to the reason that host microbiota could shape the host immune system to dictate the proinflammatory effects of other proinflammatory stimuli (128). A recent study demonstrated that the combination of remodeling of gut microbiome and microglia inhibition significantly attenuate the progression of EAU after inflammation onset (129). A potential explanation is that antigens from commensals reprogram naïve CD4^+^ T cells to the regulatory T cells lineage to restrain lymphocyte response (130). Nonetheless, it remains to be determined whether the clinical intervention targeting gut microbiome is efficacious in improving uveitis humans.

## Conclusion

Commensals are a large source of intrinsic antigens that are continuously sensed by the immune system but typically do not elicit inflammation. Since the discovery of ocular surface microbiota, their interactions with the innate immunity of the ocular surface have been explored by many researchers. Up to date, the intraocular microbiota could be considered a black box. The interior of the eye is highly vascularized and contains miscellaneous types of immune cells or immune mediators. How the intraocular microbiota and intraocular immune environment interplay to modulate inflammatory eye diseases, such as uveitis, remains an open question. Most of the microorganisms that constitute the intraocular microbiome are sparse in number, anaerobic, and extremely difficult to culture. Our laboratory has employed state-of-the-art metagenomic sequencing, combined with cultural technique and micrographical analysis to unveil the mask of intraocular microbiota, which may advance our understanding toward the mechanisms of intraocular inflammation. However, puzzles as to how commensals occupy the inside of the eye, whether the intestinal microbiota contribute to or modulate the intraocular commensals, and how intraocular commensals regulate the innate and adaptive ocular immune responses await to be answered. This process is most likely involved roles of TLRs, APCs, MHCs, and many inflammatory mediators. Further delineation of the commensal types that altered in ocular inflammatory diseases, and clarification of the question that whether the commensals as a whole or specific commensal epitopes are mainly responsible for disease progression are of significant importance to expand our knowledge of the ocular immune system and intraocular inflammation.

## Data Availability Statement

The original contributions presented in the study are included in the article/supplementary material; further inquiries can be directed to the corresponding authors.

## Author Contributions

JL and LW wrote the manuscript. SY prepared the figures. All authors contributed to the article and approved the submitted version.

## Conflict of Interest

The authors declare that the research was conducted in the absence of any commercial or financial relationships that could be construed as a potential conflict of interest.

## Abbreviations

AMD, age-related macular degeneration; DR, diabetic retinopathy; CNS, central nervous system; RGC, retinal ganglion cell; BD, Behcet’s disease; rRNA, ribosomal RNA; ITS, internal transcribed spacer; PMNs, polymorphonuclear leukocytes; ACAID, anterior chamber-associated immune deviation; cfu, colony-forming unit; HLA-B27, human leukocyte antigen-B27; MHC, major histocompatability complex; EAU, experimental autoimmune uveitis; IRBP, interphotoreceptor retinoid binding protein; TCR, T cell receptor; CF, complement factor H; ARMS2, age-related maculopathy susceptibility 2; TIMP3, tissue inhibitor of metalloproteinases-3; MMP9, matrix metallopeptidase 9; APOE, apolipoprotein E; LIPC, lipase C; CETP, cholesteryl ester transfer protein; ABCA1, ATP-binding cassette transporter; RPE, retinal pigment epithelium; LPS, lipopolysaccharide; TLR, Toll-like receptor; PAMP, pathogen-associated molecular pattern; DAMP, damage-associated molecular pattern; APC, antigen presenting cells; PRR, pattern recognition receptors; TIR, Toll/IL-1 receptor; MyD88, Myeloid differentiating factor 88; TRIF, TIR domain containing adaptor inducing interferon β; TIRAP, TIR domain-containing adaptor protein; TRAM, TRIF-related adaptor molecule; IRF, interferon regulatory factor; MAPK, mitogen-activate protein kinase; AAU, acute anterior uveitis; DC, dendritic cells; VCAM-1, vascular cell adhesion molecule-1; ICAM-1, intercellular cell adhesion molecule-1; MIP, macrophage inflammatory protein; MCP-1, monocyte chemotactic protein-1.
